# Association between autonomic dysfunction and olfactory dysfunction in Parkinson’s disease in southern Chinese

**DOI:** 10.1186/s12883-019-1243-4

**Published:** 2019-02-02

**Authors:** Xin-Yi Wang, Ying-Ying Han, Gang Li, Bei Zhang

**Affiliations:** Department of Neurology, East Hospital, Tongji University School of Medicine, 1800 Yuntai Road, Shanghai, 200123 China

**Keywords:** Parkinson’s disease, Olfactory dysfunction, Autonomic dysfunction

## Abstract

**Background:**

The aim was to investigate the autonomic dysfunction between Parkinson’s disease (PD) patients with olfactory dysfunction and PD patients without olfactory dysfunction in southern Chinese population.

**Methods:**

Fifty-six PD patients with olfactory dysfunction and 44 patients without olfactory dysfunction were included. All patients were evaluated by Sniffin’ sticks (SS-16), scales for outcomes in Parkinson’s disease-autonomic questionnaire, Hamilton anxiety rating scale and Hamilton depression rating scale

**Results:**

The score of subpart of gastrointestinal symptoms and subpart of urinary symptoms were different in two groups (gastrointestinal symptoms: *p* value: 0.024; urinary symptoms: *p* value: 0.008). As for each question items, questions 2, 8, 10, 11, 13, 14 were correlated with SS-16 scores (Question 2: *p* value: 0.013; question 6: p value: 0.006; question 8: p value: 0.025; question 10: *p* value: 0.005; question 11: p value: 0.022; question 13: *p* value: < 0.001; question 14: p value: 0.038). Question 10 and 14 were associated with olfactory dysfunction after adjusting disease duration and gender (Question 10: *p* value: 0.011, OR: 3.91; Question 14: *p* value: 0.027, OR: 3.27).

**Conclusions:**

Gastrointestinal, urinary and a part of cardiovascular symptoms of SCOPA-AUT were associated with olfactory dysfunction in PD patients.

## Background

Parkinson’s disease (PD) is a progressive movement disorder characterized by bradykinesia, rigidity and resting tremor [1]. The pathological characteristics of PD are the loss of dopaminergic neurons in the substantia nigra and abnormal α-synuclein aggregation [2]. We can divide PD symptoms into two parts, motor symptoms, such as rigidity, and non-motor symptoms such as constipation and olfactory dysfunction [3].

Olfactory dysfunction is a symptom and biomarker in PD [4, 5]. It is also a risk marker of prodromal PD [6]. Now it is taken more seriously since it is a part of peripheral symptoms. Autonomic dysfunction is also common in PD. Its symptoms can vary with different subsystems, such as constipation and cardiovascular dysautonomia [7–9]. Constipation is a risk marker of prodromal PD [6]. Both olfactory dysfunction and several autonomic dysfunctions such as constipation present prodromal biomarkers for PD with peripheral origins. But rare studies focused on their relationships in PD patients. The differences of autonomic dysfunction of PD patients with olfactory dysfunction and without olfactory dysfunction are rarely discussed. It is useful to perform early and comprehensive management in PD patients if we know better the association between olfactory function and autonomic function. In this study, we try to discover differences of several autonomic symptoms between PD patients with olfactory dysfunction and patients without olfactory dysfunction in southern Chinese PD population.

## Methods

### Study population

PD was collected from outpatients clinic of shanghai East hospital and diagnosed by movement disorder specialists based on diagnostic criteria brought up by movement disorders society (MDS) [10]. As for PD patients, Hoehn-Yahr staging and their disease duration were recorded. We excluded common secondary causes, such as inflammatory, drug-induced, vascular and toxin-induced parkinsonism. Parkinsonism with other neurodegenerative diseases, such as progressive supranuclear palsy, Wilson’s disease, cerebral-basal degeneration and multiple system atrophy was also excluded. Patients with history of operations of nose, chronic rhinitis, recent upper respiratory tract infection and smoking were also excluded. We also excluded patients with anxiety or depression using Hamilton anxiety rating scale and Hamilton depression rating scale. Patients with cognitive dysfunction assessed by Chinese version Mini-mental State Examination and Montreal Cognitive Assessment - Beijing version were ruled out. All participants signed consent forms. This study was approved by the ethic committee of shanghai East hospital.

### Olfactory tests and questionnaire evaluation

Olfactory test (Sniffin’ Sticks, SS-16) was performed to assess olfactory function through 16-item odor identification [11]. Since the threshold of SS-16 in Chinese population is 7.22 ± 1.75, we divided PD patients into two groups, PD patients with olfactory dysfunction (score of SS-16 < 5) AND PD patients without olfactory dysfunction (score of SS-16 > 9) [12]. Chinese version of Scales for Outcomes in Parkinson’s Disease-Autonomic questionnaire (SCOPA-AUT) was used to assess autonomic symptoms [13]. Hamilton anxiety rating scale and Hamilton depression rating scale were used to assess anxiety and depression. Researchers were received strict training of these scales before assessing PD patients.

### Statistics

R (version 3.5.0), stats package (version 3.5.0) was used to perform statistical analysis. t test was used to compare differences of age, disease duration and SS-16 scores between PD patients with olfactory dysfunction and patients without olfactory dysfunction. SCOPA-AUT and its subparts was assessed by multivariate regression adjusted by disease duration, Hoehn – Yahr staging, age and gender. Chi-square test was used in comparing differences of gender and 2 × 2 tables in individuals of SCOPA-AUT between two groups. Linear correlation was used to assess the correlation between the score of individuals of SCOPA-AUT and SS-16 scores. Logistic regression analysis was performed with odds ratios (OR) and 95% confidence intervals (CI) calculated. Disease duration, age and gender were also adjusted. A level of *P* < .05 was regarded as statistically significant.

## Results

In total, 56 PD patients with olfactory dysfunction and 44 PD patients without olfactory dysfunction were included in our study. There was no difference of age, gender and body mass index between PD patients with olfactory dysfunction and PD patients without olfactory dysfunction. There was no statistical difference of Hoehn – Yahr staging and disease duration between PD patients with olfactory dysfunction and PD patients without olfactory dysfunction. Of PD patients with olfactory dysfunction, the average age when they were tested was 68.29 years old and the average disease duration was 7.86 years. There was a statistical significant difference of the score of SS-16 between two groups (PD patients with olfactory dysfunction: 1.55 ± 1.04; PD patients without olfactory dysfunction: 11.93 ± 1.11, *p* value: < 0.001). However, we found statistical differences of total score of SCOPA-AUT between two groups (PD patients with olfactory dysfunction: 14.73 ± 9.05; PD patients without olfactory dysfunction: 9.75 ± 7.37, p value: 0.008). As for the score of subpart of gastrointestinal symptoms and subpart of urinary symptoms in SCOPA-AUT, there were statistical differences between two groups (Subpart of gastrointestinal symptoms: PD patients with olfactory dysfunction: 5.00 ± 4.11, PD patients without olfactory dysfunction: 3.07 ± 3.00, *p* value: 0.024; Subpart of urinary symptoms: PD patients with olfactory dysfunction: 5.38 ± 4.49, PD patients without olfactory dysfunction: 2.89 ± 3.80, *p* value: 0.008) (Table 1).

**Table 1:** Demographic data of PD patients with olfactory dysfunction and PD patients without olfactory dysfunction

	PD patients with olfactory dysfunction	PD patients without olfactory dysfunction	*p* value
	(*N* = 56)	(*N* = 44)	
Age, mean (SD)	68.29 (7.64)	67.09 (8.35)	0.463
Gender, female, N (%)	29 (51.79)	19 (43.18)	0.393
Body mass index, mean (SD)	22.19 (2.11)	22.42 (2.46)	0.631
Duration, mean (SD)	7.86 (4.40)	6.86 (5.29)	0.319
Hoehn – Yahr Staging, N (%)			
1.0	9 (16.07)	14 (31.82)	0.242
1.5	7 (12.50)	7 (15.91)	
2.0	18 (32.14)	11 (25.00)	
2.5	13 (23.21)	5 (11.36)	
3.0	5 (8.93)	6 (13.64)	
4.0	4 (7.14)	1 (2.27)	
5.0	0 (0)	0 (0)	
SS-16, mean (SD)	2.54 (1.56)	11.93 (1.11)	< 0.001
HARS, mean (SD)	1.55 (1.04)	1.39 (1.24)	0.476
HDRS, mean (SD)	1.52 (1.19)	1.32 (1.20)	0.409
SCOPA – AUT, mean (SD)*	14.73 (9.05)	9.75 (7.37)	0.008
Gastrointestinal symptoms*	5.00 (4.11)	3.07 (3.00)	0.024
Urinary symptoms*	5.38 (4.49)	2.89 (3.80)	0.008
Cardiovascular symptoms*	0.70 (1.11)	0.32 (0.86)	0.076
Skin symptoms*	2.70 (2.48)	2.66 (2.81)	0.793
Sexual symptoms*	0.50 (1.54)	0.55 (1.45)	0.804
Drug usage*	0.46 (0.66)	0.50 (1.68)	0.783

*HARS* Hamilton Anxiety Rating Scale, *HDRS* Hamilton Depression Rating Scale, *PD* Parkinson’s Disease, *SD* Standard Deviation, *SCOPA-AUT* Scales for Outcomes in Parkinson’s Disease-Autonomic questionnaire

* adjusted age, gender, disease duration and Hoehn-Yahr staging

As for each question items, the scores of question 10 “feeling that after passing urine your bladder was not completely empty” and question 14 “lightheaded when standing up” were correlated with SS-16 scores (question 10: *p* value: 0.005; question 14: *p* value: 0.038). The symptoms of question 10 and question 14 were also associated with olfactory dysfunction, with or without adjusting disease duration and gender (question 10: *p* value: 0.006, OR: 4.10, before adjustion; *p* value: 0.011, OR: 3.91, after adjustion; question 14: *p* value: 0.030, OR: 2.94, before adjustion; p value: 0.027, OR: 3.27, after adjustion). As for the correlation with SS-16 scores, the score of question 2 “saliva dribbled out of your mouth”, question 6 “strain hard to pass stools”, question 8 “difficulty retaining urine”, question 11 “the stream of urine is weak” and question 13 “have to pass urine at night” were presented these correlations (Question 2: *p* value: 0.013; question 6: *p* value: 0.006; question 8: *p* value: 0.025; question 11: *p* value: 0.022; question 13: *p* value: < 0.001). Besides, the symptom that question 6 described was associated with olfactory dysfunction before adjusting disease duration and gender (question 6: *p* value: 0.030, OR: 2.45, before adjustion) (Table 2).

**Table 2:** Association between individual items of SCOPA-AUT and olfactory dysfunction

Questions	Correlations with SS-16^a^	Correlations with individual^b^			Correlations with individual (adjusted)^b,c^		
		*p* value	OR	95% CI	*p* value	OR	95% CI
Question 1 “difficulty swallowing or have you choked”	0.306	0.950	1.05	(0.22, 5.58)	0.954	1.05	(0.21, 5.96)
Question 2 “saliva dribbled out of your mouth”	0.013	0.285	1.55	(0.70, 3.46)	0.439	1.16	(0.60, 3.18)
Question 3 “food become stuck in your throat”	0.078	0.273	2.52	(0.55, 17.82)	0.153	4.92	(0.93, 70.09)
Question 4 “full very quickly during a meal”	0.144	0.230	1.91	(0.68, 5.91)	0.264	1.87	(0.64, 6.04)
Question 5 “constipation”	0.178	0.119	1.89	(0.85, 4.25)	0.284	1.62	(6.69, 3.97)
Question 6 “strain hard to pass stools”	0.006	0.030	2.45	(1.10, 5.60)	0.066	2.24	(0.96, 5.35)
Question 7 “involuntary loss of stools”	0.194	0.996	–	–	0.996	–	–
Question 8 “difficulty retaining urine”	0.025	0.405	1.43	(0.62, 3.38)	0.521	1.33	(0.56, 3.18)
Question 9 “involuntary loss of urine”	0.705	0.182	0.49	(0.16, 1.39)	0.091	0.37	(0.11, 1.14)
Question 10 “feeling that after passing urine your bladder was not completely empty”	0.005	0.006	4.10	(1.56, 12.21)	0.011	3.91	(1.45, 12.00)
Question 11 “the stream of urine is weak”	0.022	0.318	1.73	(0.61, 5.37)	0.286	1.83	(0.62, 5.95)
Question 12 “pass urine again within 2 h of the previous time”	0.064	0.915	0.95	(0.40, 2.30)	0.682	0.82	(0.31, 2.14)
Question 13 “have to pass urine at night”	< 0.001	0.115	1.93	(0.86, 4.40)	0.144	1.85	(0.81, 4.27)
Question 14 “lightheaded when standing up”	0.038	0.030	2.94	(1.15, 8.26)	0.027	3.27	(1.20, 10.05)
Question 15 “lightheaded after standing for some time”	0.168	0.994	–	–	0.996	–	–
Question 16 “fainted in the past 6 months”	0.733	0.863	0.78	(0.03, 20.15)	0.815	1.49	(0.05, 111.72)
Question 17 “perspire excessively during the day”	0.404	0.115	0.52	(0.23, 1.17)	0.126	0.52	(0.23, 1.19)
Question 18 “perspire excessively during the night”	0.789	0.957	0.98	(0.40, 2.40)	0.963	1.02	(0.42, 2.55)
Question 19 “oversensitive to bright light”	0.490	0.867	0.90	(0.28, 3.02)	0.994	1.00	(0.29, 3.57)
Question 20 “have trouble tolerating cold”	0.799	0.690	1.20	(0.49, 3.00)	0.626	1.25	(0.51, 3.17)
Question 21 “have trouble tolerating heat”	0.852	0.498	0.73	(0.29, 1.84)	0.517	0.73	(0.28, 1.89)
Question 22 “impotent (for men)”	0.984	0.251	0.45	(0.10, 1.72)	0.221	0.41	(0.09, 1.65)
Question 23 “unable to ejaculate (for men)”	0.942	0.251	0.45	(0.10, 1.72)	0.221	0.41	(0.09, 1.65)
Question 23a “taken medication for an erection disorder (for men)”	1.000	1.000	–	–	1.000	–	–
Question 24 “vagina too dry during sexual activity (for women)”	0.463	0.163	2.81	(0.71, 14.15)	0.177	2.77	(0.68, 14.35)
Question 25 “difficulty reaching an orgasm (for women)”	0.463	0.163	2.81	(0.71, 14.15)	0.177	2.77	(0.68, 14.35)
Question 26a “use medication for constipation”	0.310	0.313	1.65	(0.64, 4.51)	0.501	1.42	(0.52, 4.06)
Question 26b “use medication for urinary problems”	0.381	0.293	3.31	(0.47, 66.00)	0.261	3.62	(0.50, 72.98)
Question 26c “use medication for blood pressure”	0.867	0.867	0.90	(0.28, 3.02)	0.877	0.91	(0.28, 3.08)
Question 26d “use medication for other symptoms”	1.000	1.000	–	–	1.000	–	–

*CI* confidence interval, *OR* odds ratio, *SCOPA-AUT* Scales for Outcomes in Parkinson’s Disease-Autonomic questionnaire, *SS-16* Sniffin’ Sticks 16 items

^a^ *p* value of the linear correlation between the score of each question and SS-16 scores

^b^ the association between each symptom described by each question and olfactory dysfunction using binomial logistic regression

^c^ adjust disease duration, gender and age

## Discussion

Our study found that differences of urinary, cardiovascular and gastrointestinal symptoms between PD patients with olfactory dysfunction and PD patients without olfactory dysfunction. To our knowledge, this is the first study to demonstrate these phenomena in PD patients in southern Chinese population.

In PD patients, constipation is the most common gastrointestinal problems. Olfactory dysfunction and constipation are both prodromal PD symptoms. There were several studies proved their associations [14, 15]. In undiagnosed individuals, hyposmia is associated with other non-motor PD symptoms, such as constipation [16]. Anosmia and constipation could progress with the primary disease process [17]. However, several researches may indicate that olfactory dysfunction and constipation may do not share the same pathogenesis. No differentially abundant gut microbes which were observed between PD and healthy individuals were replicated in nasal microbiome research [18].

The relationship between olfactory dysfunction and constipation in PD patients may not share same microbes via gut-brain axis. Besides, dopamine transporter activities are different between constipation and hyposmia in PD patients. Constipation was not associated with dopamine transporter (DAT) pathology in early drug-naïve PD patients with the help of DAT single-photon emission computed tomography (SPECT) [19]. Interestingly, idiopathic hyposmia patients who have highly chance to develop into PD were associated with the DAT pathology [20]. In PD patients, hyposmia was also correlated with striatal dopamine innervation shown by DAT binding [21]. The further relationship between olfactory dysfunction and constipation is warranted to be discovered.

Hyposmia is associated with cardiovascular dysfunction. Smell scores were associated with heart rate variability [15]. Smell scores were also associated with the increase of blood pressure of norepinephrine and dobutamine infusion tests and the uptake of cardiac ^123^I-metaiodobenzylguanidine (MIBG) [22]. Smell scores were also correlated with cardiac sympathetic degeneration presenting with early and delay heart to mediastinum ratio and the washout rate with early PD patients using MIBG uptake [23]. As for urinary problems, there was no evidence indicated that hyposmia was associated with that.

The association between olfactory dysfunction and constipation and urinary dysfunctions revealed that there was maybe more global involvement in non-motor symptoms in PD. Environmental factors, such as microbiological changes, may influence PD non-motor symptoms and pathogenesis of PD given the anatomical position of organs with olfactory function, constipation and urinary functions. More studies to discover the pathogenesis of environmental factors to PD are warranted.

The strengths of our study are that the diagnosis was based on MDS criteria. We assessed autonomic symptoms with structured scale which is widely accepted.

This study has some weakness and limitations. First, we did not perform objective clinical methods such as electrophysiology to assess autonomic symptoms. Second, we did not take relative medical history of several autonomic dysfunctions that SCOPA-AUT is not covered, such as diarrhea, dry eyes and dry mouth. These items were presented in Composite Autonomic Symptom Score (COMPASS) scale. Third, the sample of our study is small and our study was a single center study. More multicenter and larger studies are warranted.

## Conclusion

In conclusion, gastrointestinal, urinary and a part of cardiovascular symptoms of SCOPA-AUT were associated with olfactory dysfunction in PD patients. Larger and multicentral studies are warranted.

## Abbreviations

CI: Confidence interval
COMPASS: Composite Autonomic Symptom Score
DAT: Dopamine transporter
MDS: Movement disorders society
MIBG: 123I-metaiodobenzylguanidine
OR: Odds ratio
PD: Parkinson’s disease
SCOPA-AUT: Scales for Outcomes in Parkinson’s Disease-Autonomic questionnaire
SPECT: Single-photon emission computed tomography
SS-16: Sniffin’ Sticks 16
